# Prediction of Neural Diameter From Morphology to Enable Accurate Simulation

**DOI:** 10.3389/fninf.2021.666695

**Published:** 2021-06-03

**Authors:** Jonathan D. Reed, Kim T. Blackwell

**Affiliations:** ^1^Krasnow Institute of Advanced Study, George Mason University, Fairfax, VA, United States; ^2^Department of Biology, George Mason University, Fairfax, VA, United States; ^3^Department of Bioengineering, Volgenau School of Engineering, George Mason University, Fairfax, VA, United States

**Keywords:** dendritic diameter, neuron simulation, multi-compartmental model, python software, neuronal morphology, neuron reconstruction

## Abstract

Accurate neuron morphologies are paramount for computational model simulations of realistic neural responses. Over the last decade, the online repository NeuroMorpho.Org has collected over 140,000 available neuron morphologies to understand brain function and promote interaction between experimental and computational research. Neuron morphologies describe spatial aspects of neural structure; however, many of the available morphologies do not contain accurate diameters that are essential for computational simulations of electrical activity. To best utilize available neuron morphologies, we present a set of equations that predict dendritic diameter from other morphological features. To derive the equations, we used a set of NeuroMorpho.org archives with realistic neuron diameters, representing hippocampal pyramidal, cerebellar Purkinje, and striatal spiny projection neurons. Each morphology is separated into initial, branching children, and continuing nodes. Our analysis reveals that the diameter of preceding nodes, Parent Diameter, is correlated to diameter of subsequent nodes for all cell types. Branching children and initial nodes each required additional morphological features to predict diameter, such as path length to soma, total dendritic length, and longest path to terminal end. Model simulations reveal that membrane potential response with predicted diameters is similar to the original response for several tested morphologies. We provide our open source software to extend the utility of available NeuroMorpho.org morphologies, and suggest predictive equations may supplement morphologies that lack dendritic diameter and improve model simulations with realistic dendritic diameter.

## Introduction

Neuronal morphology is the foundation for computational models which integrate molecular and cellular processes to understand brain function and behavior (Fan and Markram, 2019). Realistic neural modeling requires comprehensive biological description of neurons and synapses (D’Angelo et al., 2013), while simulation of neural networks may utilize heterogenous neural populations (Einevoll et al., 2019), including heterogeneity in neuronal morphology. Individual neuron morphologies are the basis for computational simulation of neural response, branching (Cuntz et al., 2007, 2010; Donohue and Ascoli, 2008), and growth (Koene et al., 2009). Neuron model simulations have shown that neuronal morphology may affect firing response (Chen, 2010), either due to branching complexity (van Elburg and van Ooyen, 2010), channel arrangement (Zhou et al., 2015), or dendritic length (Li et al., 2015).

The diameter of neuronal branches is particularly important for controlling the flow of ionic current and signaling molecules, and thus is critically important for simulating neuron electrical activity. In his landmark study (Rall, 1962), Rall showed that if the diameter of the parent branch (raised to the 3/2 power) was equal to the sum of diameters of the child branches (raised to the 3/2 power) then the two child branches could be replaced by an equivalent cylinder (providing a few other conditions were met). Since then, reconstructions of neuronal morphology have shown that this diameter relationship does not always hold (Cullheim et al., 1987; Scorcioni et al., 2004; Krichmar et al., 2006; Zomorrodi et al., 2010; Kubota et al., 2011), and dendrites taper between branch points in some neuron types. Nonetheless, changes in dendritic branch diameter can maximize current transfer (Bird and Cuntz, 2016) and influence Ca^2+^ dynamics (Anwar et al., 2014) or other second messengers (Luczak et al., 2017). Conversely, resources and second messengers can influence dendritic growth. For example, diameter is observed to change in response to cell growth (Mironov et al., 2016). In addition, diameter of neural branches is observed to change dynamically, due to competition of resources between local branches (Hjorth et al., 2014) or after high-frequency stimulation (Chéreau et al., 2017). Thus, understanding neuron function from morphology requires measures of diameter.

Currently, NeuroMorpho.org remains the largest online repository to access neuron morphological data, with over 140,000 neuron morphologies from numerous brain regions, neuron types, and species (Ascoli, 2015; Ascoli et al., 2017). Though an insightful tool, NeuroMorpho.org contains many neuron morphologies that lack measurement of dendritic diameters. NeuroMorpho.org reviews certain aspects of morphology for all submitted archives (Parekh et al., 2015); however, many neuron morphologies contain dendritic diameters that lack dendritic tapering or branching asymmetry (Brown et al., 2008), or contain identical diameters where variability is expected (Anwar et al., 2014).

One possibility to obtain realistic dendritic diameters is to create equations to predict diameter from other morphological features. In essence, is it possible to extend Rall’s seminal work and use relationships among other morphological features to predict the diameter of dendritic branches? A previous study describes an equation to estimate diameter, which included total dendritic length, though is limited to a single neuronal morphology (Lindroos et al., 2018). Expanding this approach to multiple archives and cell types would enhance the use of neuron morphologies with realistic dendritic diameter for model simulation.

Our study derives equations to predict dendritic diameter from other morphological features for three neuron types; hippocampal pyramidal, cerebellar Purkinje, and striatal spiny projection neurons (SPNs). We demonstrate that Parent Diameter (PD) is strongly correlated to Child Diameter across multiple cell types, particularly for hippocampal pyramidal cells. The primary (initial) nodes, which begin dendritic processes, and nodes directly after bifurcation (branching children) require a combination of morphological features to predict diameter, such as path length to soma, total dendritic length, and longest path to terminal end, though this varies by cell type. Simulations reveal membrane potential responses for passive models with predicted diameters were similar to those of models with original diameters, including morphologies not used to derive the equations. Our predictive equations may extend utility of available morphologies on NeuroMorpho.org with realistic dendritic diameters.

## Materials and Methods

### Summary

Several archives from NeuroMorpho.org were selected as suitable for predictive diameter equations. Each reconstruction contains a collection of points (nodes) to describe neuronal morphology. Each node acts as a boundary for compartments or segments, which are cylindrical-like spaces useful for model simulation of membrane potential in response to current injection or synaptic input. Measures describing each node (features) within neuron morphologies were compared to node diameter, using graphical and statistical approaches, to reveal possible predictive relationships. Multiple regression using a combination of morphological features produced equations to predict node diameter. New morphologies with predicted diameters were created and compared to original morphologies, and a subset of the neurons were simulated to assess differences in passive response between original and predicted diameters.

### NeuroMorpho.Org Archive Selection

**FIGURE 1:**
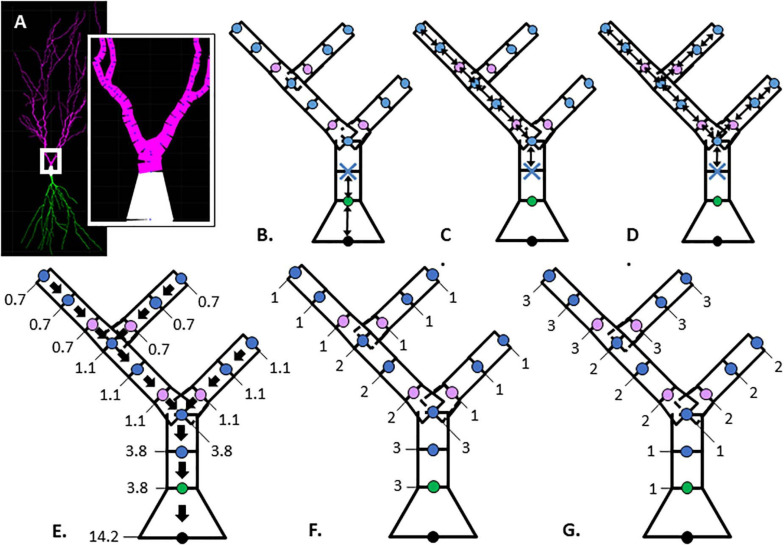
Illustration of morphological features. **(A)** Hippocampal CA1 pyramidal neuron visualized with Cvapp from NeuroMorpho.org (NMO_00817, left panel); start of apical branch (right panel) with compartments (magenta) between individual nodes (blue dots). **(B–G)** Simplified dendritic morphology of apical branch to demonstrate morphology feature values for each node. Nodes (points) classified as soma (black), initial (green), continuing (blue), and branch children (lavender). Further feature description in Table 2. **(B–D)** Arrows indicate path distance between nodes used to calculate feature values originating at the first continuing node, marked with blue X. **(B)** Path from soma. **(C)** Longest Path to Terminal End. **(D)** Total Dendritic Length Rooted at Node. **(E)** Parent Diameter; arrows point to parent node and values provide the node diameter (μm). **(F, G)** Feature values indicate node positional relationship in branch arrangement within morphology. **(F)** Terminal Degree. **(G)** Initial Branch Order.

NeuroMorpho.org metadata search and morphology inspection provided initial archives for consideration toward predictive diameter equations. Using metadata search, we selected certain morphological- and reconstruction-specific aspects with emphasis on dendritic representation (Parekh et al., 2015). In *Animal: Species*, we selected only mouse or rat neuron morphologies. In *Completeness*, we specified our search to only include (a) *Morphological Attributes*: morphologies with diameter, either 2-dimensional or 3-dimensional, and with or without angles, (b) *Structural Domain*: morphologies with dendrites, soma, and with or without axon, and (c) *Physical Integrity: search by dendrites*: morphologies with “*complete*” dendrites, or non-fragmented/non-truncated processes. In *Experiment: Experimental Condition*, we specified control. Following metadata search, archives were retained if available brain regions or similar cell types contain at least two separate archives, and if each archive contains more than a single morphology. Subsequent visual inspection of the remaining 35 archives (totaling 790 morphologies) revealed some morphology issues that forced exclusion from analysis. These issues included identical diameter across all dendritic nodes and dendritic processes with extreme shifts in the *z*-plane. From the seven selected archives (Table 1), morphological features were obtained to relate node measures to node diameter within morphology files. Thus, we extracted morphology feature values to describe each dendritic node (Table 2 and Figure 1).

**TABLE 1:** Selected NeuroMorpho.org archives for predictive diameter equations.

Archive	Brain region	Cell type	Morphology files	Dendritic nodes	Mean nodes per cell
Groen (Groen et al., 2014)	Hippocampus	CA1 Pyramidal	12	172023	14335
Jaffe (Chitwood et al., 1999)	Hippocampus	CA3 Pyramidal	5	14507	2901
Nedelescu (Nedelescu et al., 2018)	Cerebellum	Purkinje	30	46748	1558
Dusart (Chen et al., 2013)	Cerebellum	Purkinje	6	19664	3277
Luebke (Goodliffe et al., 2018)	Striatum	D1R SPN	14	25954	1853
Luebke (Goodliffe et al., 2018)	Striatum	D2R SPN	14	26068	1862
Lai (Chen et al., 2014)	Striatum	SPN	10	58629	15862

*As described in section “Results,” hippocampal CA3 and CA1 pyramidal cells were combined for subsequent analysis to develop predictive equations.*

**TABLE 2:** Description of morphological features.

Acronym	Feature	Description
D	*Diameter*	Node diameter as defined by 2× radius within SWC morphology file.
PD	*Parent Diameter*	Diameter of the previous node in path. Dendritic nodes which directly stem from soma, i.e., “initial nodes,” have the soma as the parent node.
IB	*Initial Branch Order*	Initial nodes initialized as 1. Traversing downstream away from soma, each branching node increases value by 1.
TD	*Terminal Degree*	Terminal nodes initialized as 1. Traversing upstream toward soma, value increases at each branching node as the sum of terminal degree of the two downstream (child) nodes.
PS	*Path from Soma*	Summed path distance from soma traversing downstream toward selected node.
LP	*Longest Path to Terminal End*	Summed path distance from selected node and traversing downstream toward terminal node in single path with greatest distance.
TL	*Total Dendritic Length Rooted at Node*	Summed path distance stemming from selected node and traversing downstream toward all terminal nodes in path.

*Individual node measures calculated from SWC neuron morphologies. Morphological features are explained visually in Figure 1.*

### Feature Prediction and Modeling Dendritic Diameter

Several statistical tools were used to relate morphological features to node diameter. Graphical analysis and Pearson’s coefficient of determination (*R*^2^) were used to identify features that were correlated with Diameter but not with each other. The adjusted *R*^2^ from Multiple Linear Regression using an Ordinary least squares (OLS) was used to automate selection of features that account for a high proportion of variance in predicting node diameter. First, we calculated the adjusted *R*^2^ between Diameter and each feature. Second, we calculated the adjusted *R*^2^ of Diameter + an additional feature, in order from highest to lowest feature adjusted *R*^2^. If the adjusted *R*^2^ for the pair exceeded the *R*^2^ for each single feature (by a small improvement constant) that pair was added to the list of candidate models. Third, we calculated the adjusted *R*^2^ of other feature combinations, and added that combination to the list if the adjusted *R*^2^ for the pair exceeded the *R*^2^ for each single feature and was better than the prior best adjusted *R*^2^. Note that the same results were obtained using an improvement constant of 0.001 or 0.02.

The final model fit was determined using Multiple Linear Regression using an OLS model applied to the training set, and the single or pair of features producing the best adjusted *R*^2^ as determined using all the data. Note that if the regression includes an intercept, the Pearson’s *R*^2^ will be identical to the model *R*^2^; however, our final regressions did *not* include an intercept in these final model fits. For each cell type, archive morphologies were randomly separated into a training set (Train), to create predictive equations, or a testing set (Test), to predict diameters of morphologies independent from those used for predictive equations. Original and predicted diameters were compared for morphologies in the training set and morphologies in the testing set separately. When comparing predicted and original diameters, goodness-of-fit was calculated using Pearson’s coefficient of determination (*R*^2^) for each neuronal morphology and averaged across morphologies.

We used the regression equations to create the morphologies with predicted diameters. The process begins at initial nodes and uses the soma diameter and selected features in predictive equations. Traversing away from the soma, all subsequent branch children and continuing nodes use the predicted PD instead of original PD. That Predicted PD, in addition to selected features in the predictive equations, then determine predicted node diameter until all dendritic nodes have predicted diameter.

### Simulation

Simulation of individual neuron responses to somatic current injection was used to assess functional quality of predicted diameter. To verify our model equations, we simulated one morphology from each of the three selected cell types: hippocampal pyramidal (NMO_35137), striatal SPN (NMO_33253), and cerebellar Purkinje (NMO_10073) cells. Two additional morphologies separate from the training or testing set were simulated to validate the model equations. One hippocampal CA1 morphology was selected which had published passive morphology simulations (NMO_00886, Golding et al., 2005) and one striatal SPN was selected to compare predicted diameters from our equations to previously reported diameter equations (NMO_08390, Lindroos et al., 2018). The selected hippocampal CA1 pyramidal cell (NMO_00886) was modified to a 3-point soma for simulation in Moose^1^. The same membrane and cytosolic parameters (RM = 1.6 Ω m^2^, CM = 0.0186 F/m^2^, and RA = 1.98 Ω m) were used for simulations as previously reported (Golding et al., 2005), with the exception of the independent striatal SPN (RM = 8 Ω m^2^, CM = 0.01 F/m^2^, and RA = 0.15 Ω m; NMO_08390; Lindroos et al., 2018). Both a brief (1 ms, 1.5 nA) and a prolonged (800 ms, 30 pA) current injection at the soma were used to evaluate time constant (τ, fit to double exponential) and steady state voltage response (ΔV), respectively. We also simulated the response to synaptic input, which had a conductance of 10 pS, rise time constant of 1 ms, decay time constant of 5 ms, and reversal potential of 5 mV. We compared the predicted and original values of time constant and steady state as the normalized difference (ratio): the difference between time constant or steady state of the predicted and original morphology divided by the time constant or steady state of the original morphology. We similarly compared the peak synaptic depolarization between predicted and original morphologies as a normalized difference.

### Software Implementation

Several open-source Python programs were created and are available from^2^. *morph_feature_extract.py* (Python 2.7) uses the Python 2 package *btmorph*^3^ to calculate morphological features from SWC format morphologies. *morph_feature_analysis.py* (Python 3.6) graphs morphological features versus diameter, and uses *statsmodels*^4^ to perform statistical analysis on extracted features to create predictive equations for node diameter. *shape_shifter.py* (Python 3.6) utilizes predictive equations to create new morphologies with predicted diameters from original morphologies. Individual neurons, both original and with predicted diameters, are simulated using Moose (see text footnote 1). The Python scripts used for the Moose simulations are available in the ShapeShifter respository.

## Results

### Morphological Features Describe Node Diameter

In order to derive equations to predict diameter, we identified three cell types consisting of six separate archives: hippocampal pyramidal (Chitwood et al., 1999; Groen et al., 2014), cerebellar Purkinje (Chen et al., 2013; Nedelescu et al., 2018), and striatal SPNs (Chen et al., 2014; Goodliffe et al., 2018; Table 1). From 121,544 morphologies available on NeuroMorpho.org (ver. 7.9), metadata search parameters identified 3,463 morphologies (2.85%) from selected criteria. Archive exclusion further decreased the number to 35 potential archives totaling 790 morphologies before visual inspection of morphology. Multiple morphological features were calculated for each node in archive morphologies for assessment in predicting node diameter (Table 2 and Figure 1).

### Parent Diameter as Predictor of Diameter

**FIGURE 2:**
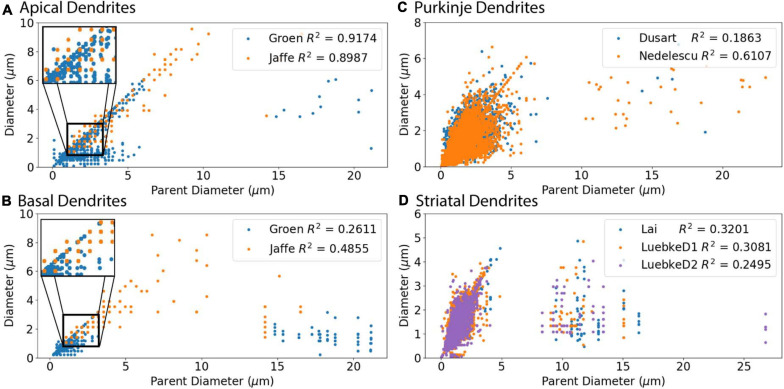
Parent Diameter predicts diameter for multiple neuron types. *R*^2^ as Pearson’s coefficient of determination for each archive. **(A–D)** A majority of nodes display a linear relationship between Parent Diameter and diameter, with a subset of nodes with large Parent Diameter departing from the relationship. **(A)** Apical dendrites of hippocampal pyramidal morphologies, with inset near origin showing the discretization of diameter values; Groen (CA1) with 130,070 nodes, Jaffe (CA3) with 8,483 nodes. **(B)** Basal dendrites of hippocampal pyramidal morphologies, with inset near origin; Groen (CA1) with 41,953 nodes, Jaffe (CA3) with 6,024 nodes. **(C)** Cerebellar Purkinje morphologies; Dusart with 19,664 nodes, Nedelescu with 46,748 nodes. **(D)** Striatal SPN morphologies; Luebke D1R (LuebkeD1) with 25,954 nodes, Luebke D2R (LuebkeD2) with 26,068 nodes, and Lai with 58,629 nodes.

We first evaluated the correlation of PD to diameter, because dendrites tend to decrease slowly in diameter. Figure 2 shows that PD is moderately correlated to diameter for all cell types tested. Analysis of hippocampal CA3 and CA1 pyramidal archives revealed that both are moderately correlated to PD (Figure 2), with almost identical regression lines, despite having different distributions of features (Supplementary Figures 1,2). Based on this similarity, we combined these two data sets into a single hippocampal pyramidal cell group for the remainder of analyses. Accounting for all dendritic nodes in the morphology, apical (*R*^2^ = 0.899, 0.917) and basal (*R*^2^ = 0.486, 0.261) dendrites of hippocampal pyramidal cells, for the Jaffe and Groen archives, had moderate correlation to PD, as did striatal SPNs (*R*^2^ = 0.320, 0.308, 0.250), for the Lai, LuebkeD1, and LuebkeD2 archives. Cerebellar Purkinje cells (*R*^2^ = 0.186, 0.611) for the Dusart and Nedelescu archives have diverse correlations to PD. Moderate correlation to PD indicates this feature as a potential predictor of node diameter across different cell types, particularly for apical dendrites of hippocampal pyramidal cells. To further ascertain whether other features could be used to predict diameter independent of archive, we calculated the correlation between diameter and features for each archive, and distribution of feature values for each archive. Table 3 shows that, for the striatum and hippocampus, the correlation of diameter to other features are similar across archives, and Supplementary Figure 3 shows that the feature distributions are similar for the striatal archives. The difference in *R*^2^ between cerebellum archives averages ∼0.3 across features and the diameter distributions are quite different (Supplementary Figure 4), suggesting that additional archives may be needed to create equations that will generalize across novel archives. The cause of this disparity is unclear, but could include mouse strain, age, sex, cerebellar subregion, slice thickness, staining method, or other aspects not recorded by NeuroMorpho.org.

**TABLE 3:** Correlation between morphological features and diameter by archive.

	Cerebellum		Striatum			Hippocampus, basal		Hippocampus, apical	
Correlation to diameter	Dusart	Nedelescu	Lai	Luebke D1	Luebke D2	Groen	Jaffe	Groen	Jaffe
Path distance	0.0694	−0.2173	0.2685	0.1842	0.1849	−0.4434	−0.368	−0.2275	−0.3439
Path to end	0.3211	0.7217	0.3457	0.0985	0.0997	0.4301	0.3929	0.8248	0.625
Total dendritic length	0.3418	0.6729	0.4931	0.1479	0.1769	0.5557	0.6892	0.8301	0.785
Node order	0.0828	0.3241	0.0649	−0.1032	−0.1549	−0.4662	−0.2508	−0.0759	−0.3589

### Diameter Predictions Differ With Node Classification

**FIGURE 3:**
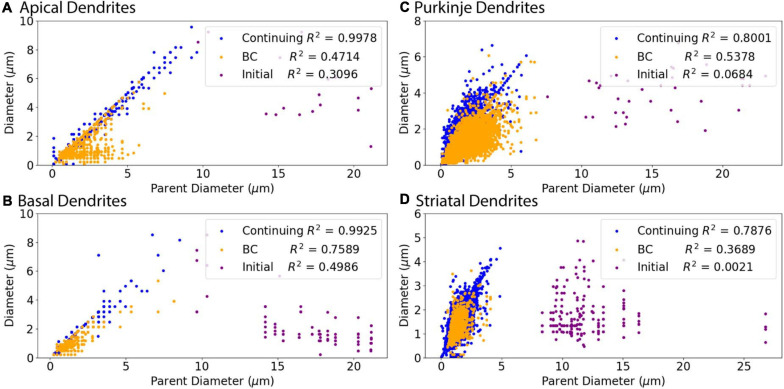
Relationship of Parent Diameter to diameter differs for Initial nodes. Note that the data points are identical to those in Figure 2, but color coded according to node type instead of archive. *R*^2^ as Pearson’s coefficient of determination for each node type. **(A–D)** Nodes separated into initial, branching children (BC), and continuing nodes. Branching children and continuing nodes demonstrate strong linear relationship between Parent Diameter and diameter, which is lacking for initial nodes. Node classification explained in Figure 1. **(A)** Apical dendrites of hippocampal pyramidal morphologies have 138,536 continuing nodes, 17 initial nodes, and 1,510 branching children nodes. **(B)** Basal dendrites of hippocampal pyramidal morphologies have 47,899 continuing nodes, 78 initial nodes, and 776 branching children nodes. **(C)** Cerebellar Purkinje morphologies have 66,376 continuing nodes, 36 initial nodes, and 13,200 branching children nodes. **(D)** Striatal SPN morphologies have 110,455 continuing nodes, 196 initial nodes, and 1,784 branching children nodes.

We subdivided the nodes into different classes to account for the 3/2 power rule for branch points. Thus, one node type is branching children, which directly stem from branch nodes. In addition, we noticed a small subset of nodes with large PD, which departed from the main linear relationship to PD for all cell types (Figure 2). The large PD of these nodes suggests that these nodes may be the initial dendritic nodes that directly stem from the soma. Thus another node type is initial node. The remaining nodes are the third class: continuing nodes. We reevaluated the correlation between PD and Diameter separately for the three classes of nodes: initial, branching children, or continuing nodes. Figure 3 verifies that initial nodes are indeed the nodes with large PD, and these nodes do not exhibit a linear relationship. On the other hand, the branching children and continuing nodes retain similar linear relationship and result in moderate to strong correlation with PD (Figure 3). Note that the correlation between Diameter and PD for branching children was not improved by raising these values to the 3/2 power. Due to the difference in node type, all subsequent analyses were performed separately for initial, branching children, and continuing nodes.

**FIGURE 4:**
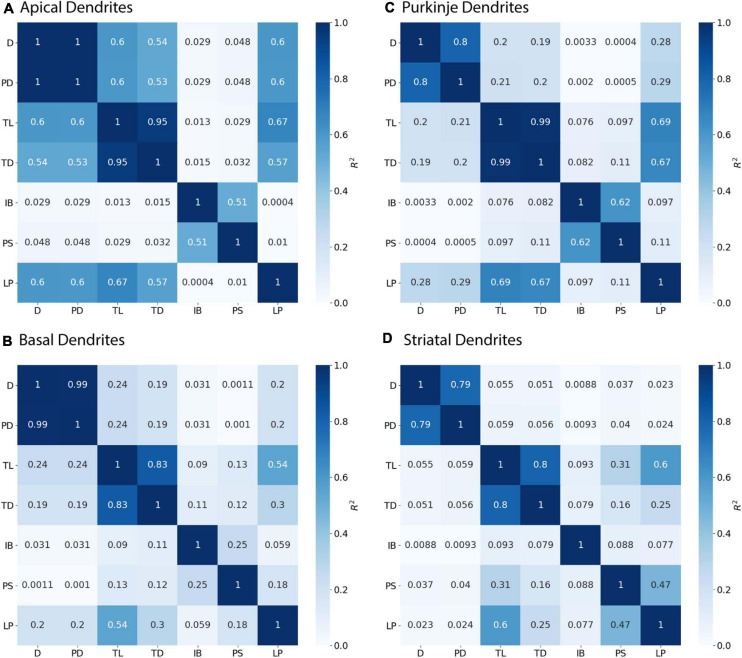
Parent Diameter predicts diameter for continuing nodes. *R*^2^ as Pearson’s coefficient of determination. TL, Total Dendritic Length Rooted at Node; TD, Terminal Degree; IB, Initial Branch Order; PD, Parent Diameter; PS, Path from Soma; LP, Longest Path to Terminal End; and D, Diameter. Morphology feature descriptions from Table 2. **(A–D)** Diameter of continuing nodes have strong correlation with PD across morphologies. Apical dendrites of hippocampal pyramidal cells have multiple features with moderate to strong correlation with diameter. Remaining cell types have low correlation with other features.

Figure 3 shows that diameter of continuing nodes is highly correlated to PD across cell types. Strong correlation in apical dendrites (*R*^2^ = 0.998) and basal dendrites (*R*^2^ = 0.993) of hippocampal pyramidal cells, cerebellar Purkinje cells (*R*^2^ = 0.800), and striatal SPNs (*R*^2^ = 0.788) indicate PD as a strong predictor of continuing node diameter (Figure 4). No additional features improved the prediction of diameter for continuing nodes. We found node diameter was equal to PD for the vast majority of continuing nodes in hippocampal pyramidal cells, consisting of 98.2% of apical dendrites, and 97.3% of basal dendrites. Node diameter was equal to PD for a moderate number of continuing nodes in cerebellar Purkinje cells (51.2%) and striatal SPNs (65.9%).

**FIGURE 5:**
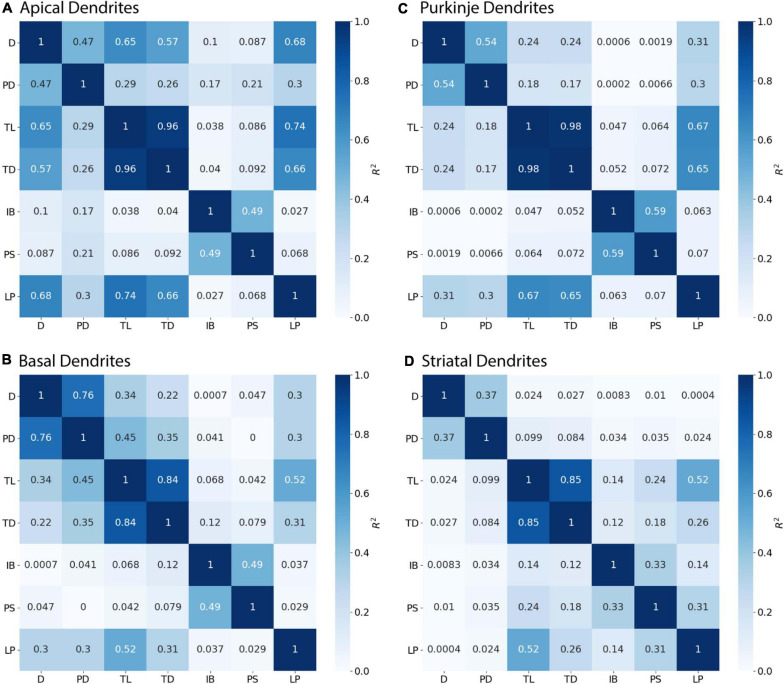
Branching Children require Parent Diameter in combination with other features to predict diameter. *R*^2^ as Pearson’s coefficient of determination. **(A–D)** Diameter of branching children have moderate to strong correlation with Parent Diameter (PD) across morphologies. Better model fits to diameter were obtained by adding select features with PD: Longest Path to Terminal End (LP) for apical dendrites of hippocampal pyramidal cells, Path to Soma (PS) for basal dendrites of hippocampal pyramidal cells, and Total Dendritic Length (TL) for cerebellar Purkinje cells.

Branching children require additional morphological features in combination with PD to predict their diameter. Branching children demonstrate lower correlation to PD than observed with continuing nodes (Figure 5). Moderate correlation in apical dendrites (*R*^2^ = 0.471) and basal dendrites (*R*^2^ = 0.759) of hippocampal pyramidal cells, cerebellar Purkinje cells (*R*^2^ = 0.538), and striatal SPNs (*R*^2^ = 0.369) indicate PD is still a predictor of diameter of branching children (Figure 5). Because other features were correlated with diameter, we used multiple linear regression to select additional features that were both predictive of diameter and improved the overall model adjusted *R*^2^. When combined with PD, the OLS model produced a good fit by including Longest Path to Terminal End for apical dendrites of hippocampal pyramidal cells (adj *R*^2^ = 0.915), Path to Soma for basal dendrites of hippocampal pyramidal cells (adj *R*^2^ = 0.934) and Total Dendritic Length for cerebellar Purkinje cells (adj *R*^2^ = 0.883). We tested whether using the non-linear 3/2 power rule could improve the correlations for branching children. The correlation between PD raised to the 1.5 and the *sum* of child diameters raised to the 1.5 was quite high (between 0.64 and 0.93 for all but the Groen Apical dendrites). However, the predictive model was not improved by using the 3/2 power rule because the model must predict individual child diameters, not the sum of child diameters. Graphs of diameter versus the selected feature were used to visually verify the lack of other non-linear relationships between feature values and diameters for branching children.

**FIGURE 6:**
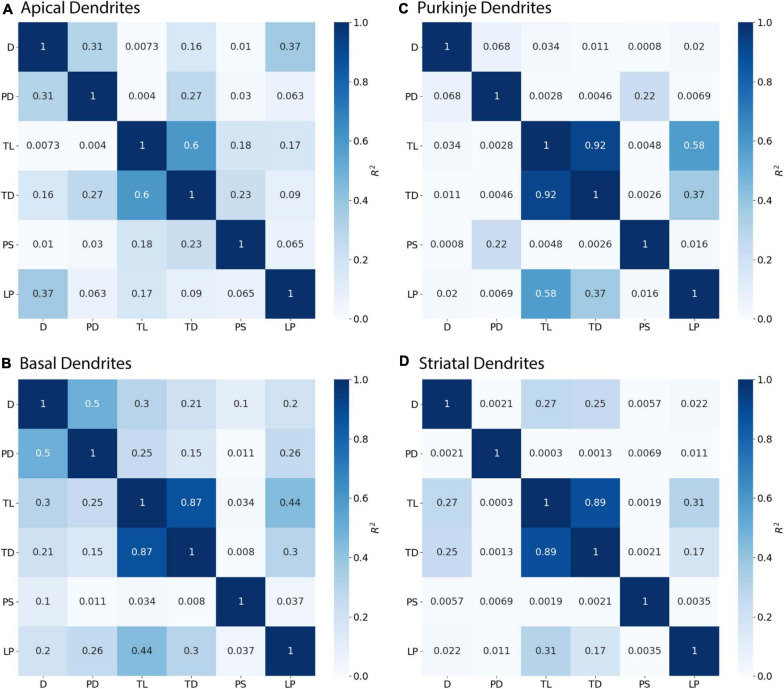
Initial Nodes require multiple features to predict diameter. *R*^2^ as Pearson’s coefficient of determination. **(A–D)** Diameter of initial nodes have moderate correlation with Parent Diameter (PD) for apical and basal dendrites of hippocampal pyramidal cells, and low correlation for cerebellar Purkinje cells and striatal SPNs. Stronger correlation to diameter for hippocampal dendrites was obtained by adding select features with PD. Diameter was correlated with Longest Path to Terminal End (LP) for apical dendrites of hippocampal pyramidal cells, and Path to Soma (PS) for basal dendrites of hippocampal pyramidal cells. Diameter was correlated with Total Dendritic Length (TL), but not PD, for striatal SPNs.

Initial nodes also require a combination of morphological features to predict diameter. Diameter of apical dendrites (*R*^2^ = 0.310) and basal dendrites (*R*^2^ = 0.499) of hippocampal pyramidal cells have lower correlation to PD (soma diameter), with even lower correlation in cerebellar Purkinje cells (*R*^2^ = 0.068) and striatal SPNs (*R*^2^ = 0.002; Figure 6). We used multiple linear regression to select features predictive of diameter. The OLS model indicated moderate to good fit by using PD with Longest Path to Terminal End for apical dendrites of hippocampal pyramidal cells (adj *R*^2^ = 0.722), and PD with Path to Soma for basal dendrites of hippocampal pyramidal cells (adj *R*^2^ = 0.725). Initial nodes of striatal SPNs did not use PD and instead the best model used Total Dendritic Length and Longest Path to Terminal End (adj *R*^2^ = 0.813). The best model for cerebellum used PD alone (adj *R*^2^ = 0.888). Graphs of diameter versus feature value were used to visually verify the lack of non-linear relationships between the feature values and diameters for initial nodes.

### Original Initial Diameters Improve Hippocampal Pyramidal Predictions

**FIGURE 7:**
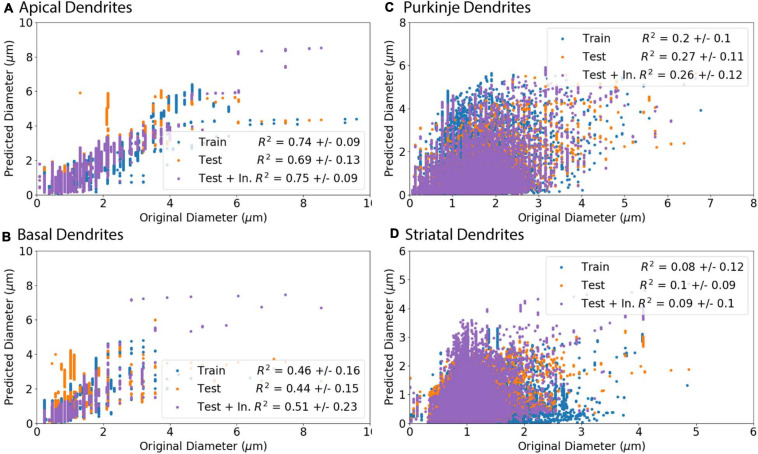
Predicted Diameters match original Diameters for select archives. **(A–D)** Morphologies were separated into a training set (Train) to create predictive equations and verified with separate testing set (Test). As initial nodes were not predicted as well as branching children or continuing nodes, we created an additional set of morphologies from the test set that used the original diameter of initial nodes and then predicted diameter for remaining branching children and continuing nodes (Test + In.). *R*^2^ is the averaged Pearson’s coefficient of determination *R*^2^ calculated across morphologies in either the training or testing set. Predictions which include original initial diameters have higher correlation with original diameters for apical dendrites (*R*^2^ = 0.75) and basal dendrites (*R*^2^ = 0.51) of hippocampal pyramidal cells, though did not improve correlation for cerebellar Purkinje cells (*R*^2^ = 0.26) or striatal SPNs (*R*^2^ = 0.09). **(A, B)** Apical and basal dendrites of hippocampal pyramidal with 8 in the training set and 9 in the testing set. **(C)** Cerebellar Purkinje morphologies with 18 in the training set and 18 in the testing set. **(D)** Striatal SPN morphologies with 19 in the training set and 19 in the testing set.

To evaluate the ability to predict diameter, we separated morphologies into a training set (Train), to derive model equations, and a testing set (Test), to predict diameter independent of morphologies used to derive the predictive equations. The model adjusted *R*^2^ (adj *R*^2^) in Table 4 shows goodness of fit for the training set morphologies. The equations in Table 4 solely require the soma diameter and other morphological features (Table 2) to predict diameter across morphology, and do not rely on original dendritic diameters. The correlation between original and predicted diameter is shown in Figure 7.

**TABLE 4:** Predictive diameter equations.

	Initial nodes	Branch children	Continuing nodes
*Hippocampal pyramidal Apical dendrites*	0.0755 × PD + 0.0056 × LP (adj *R*^2^ = 0.7216)	0.2598 × PD + 0.0034 × LP (adj *R*^2^ = 0.9151)	0.9968 × PD (adj *R*^2^ = 0.9993)
*Hippocampal pyramidal Basal dendrites*	−0.5964 × PD + 0.3535 × PS (adj *R*^2^ = 0.7246)	0.6351 × PD + 0.0033 × PS (adj *R*^2^ = 0.9340)	0.9926 × PD (adj *R*^2^ = 0.9984)
*Cerebellar Purkinje (apical like)*	0.2331 × PD (adj *R*^2^ = 0.8879)	0.6842 × PD + 0.6842 × TL (adj *R*^2^ = 0.8934)	1.0121 × PD (adj *R*^2^ = 0.9555)
*Striatal SPN (basal like)*	0.00114 × TL + 0.00713 × LP (adj *R*^2^ = 0.8126)	0.921 × PD (adj *R*^2^ = 0.915)	0.9834 × PD (adj *R*^2^ = 0.9753)
*Striatal SPN, Lai*	0.250 × TD (adj *R*^2^ = 0.786)	0.861 × PD (adj *R*^2^ = 0.967)	0.997 × PD (adj *R*^2^ = 0.999)
*Striatal SPN, Luebke*	0.003 × TL (adj *R*^2^ = 0.75)	0.934 × PD (adj *R*^2^ = 0.907)	0.976 × PD (adj *R*^2^ = 0.962)

*TL, Total Dendritic Length Rooted at Node; PD, Parent Diameter; PS, Path from Soma; and LP, Longest Path to Terminal End. Morphology feature acronyms from Table 2. adj *R*^2^ as measure of training set fit of predicted diameters to original diameters across morphologies.*

Predicted diameters of testing set moderately match original diameters of apical dendrites (*R*^2^ = 0.69) and basal dendrites of hippocampal pyramidal cells (*R*^2^ = 0.44) and have low correlation for cerebellar Purkinje cells (*R*^2^ = 0.27) and striatal SPNs (*R*^2^ = 0.1; Figure 7). Pearson’s coefficient of determination *R*^2^ is calculated for each dendritic tree, and then averaged to obtain a mean *R*^2^ separately for training set and testing set. As the prediction of diameter for initial nodes is weak compared to branch children and continuing nodes, we used the original diameter of initial nodes (original initial diameter) to predict new diameters for remaining branch children and continuing nodes. The predictions using the original initial diameters (Test + In. in Figure 7) had greater correlation to original diameters for apical dendrites (*R*^2^ = 0.75) and basal dendrites (*R*^2^ = 0.51) of hippocampal pyramidal cells. Using the original initial diameters did not significantly improve correlation to original diameters for cerebellar Purkinje cells (*R*^2^ = 0.26) or striatal SPNs (*R*^2^ = 0.09; Figure 7).

**FIGURE 8:**
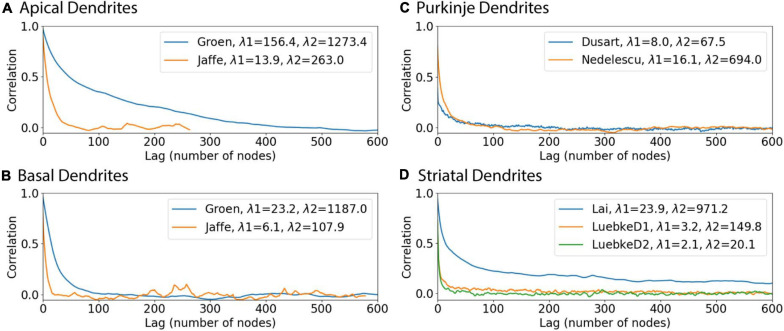
Autocorrelation functions illustrate why predictions of diameter are better for some dendrites. In each archive, the autocorrelation was averaged across morphologies to reduce noise, and then the average autocorrelation was fit to a double exponential decay. **(A)** Hippocampal apical dendrites, **(B)** Hippocampal basal dendrites, **(C)** Purkinje dendrites, **(D)** Striatal dendrites. The smaller space constant, λ1, of hippocampal apical dendrites is larger than that of other dendrites. The larger space constant, λ2, of both apical and basal hippocampal dendrites is larger than that of striatal and cerebellar dendrites.

We investigated two sources that may contribute to the discrepancy between correlation with predicted diameters and the original correlations. We considered whether a difference between archives could cause prediction errors by repeating the model fits separately for individual archives. The fit to one of the striatal archives was improved, while the fits for all the other archives were either worse, or similar. The parameters for the Lai archive alone and the two Luebke archives together show that, for branch children and continuing nodes, the parameter values for the three archives together are between the parameter values for the separated archives. For the initial nodes, different features were used, thus the parameters cannot be compared. Then we evaluated the auto-correlation for each archive. The autocorrelation differs from the correlations shown in Figures 2, 3 in that it evaluates the correlation between the diameters of nodes *separated by multiple nodes*. Figure 8 shows that the autocorrelation decay constants are larger for hippocampal apical dendrites than for other dendrites, and smallest for the Luebke archives. Since the goal is to predict all diameters, starting from the soma (or from the initial nodes), the fast decay of the autocorrelation (smaller space constant is worse) together with the maximum correlations to diameter from Figures 4, 5 (larger is better) jointly determine the quality of the predictions. In summary, predicted diameters are moderately to strongly correlated with original diameters of hippocampal pyramidal cells and cerebellar Purkinje cells, but do not capture diameter variations of striatal SPNs as well. However, a more functional test of diameter predictions is to simulate the membrane potential response of neurons, as the purpose of predicting diameter is to expand the number of morphologies that could be used in model simulations.

### Predictions With Original Initial Diameters Improve Simulation Passive Response

**FIGURE 9:**
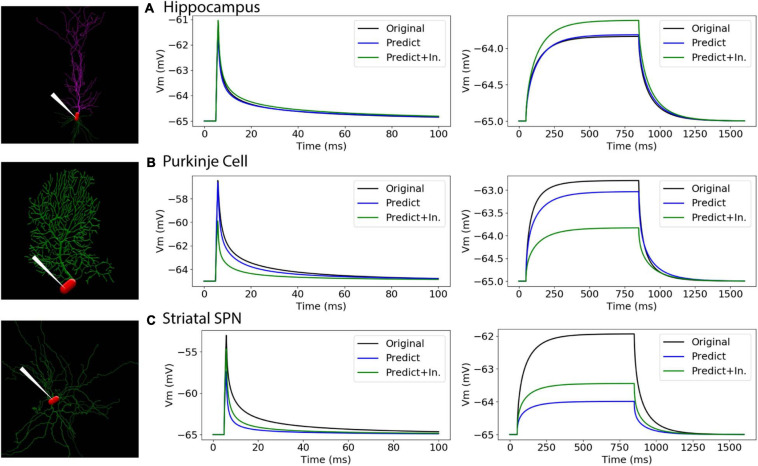
Passive response is similar when predicted diameters are correlated with original diameters. **(A–C)** Simulation strategy, with location of current injection and voltage recording at the soma compartment (left panel); 1 ms (center) and 800 ms (right) current injection. **(A)** Hippocampal CA1 pyramidal cell (NMO_35137), with apical and basal dendrites. **(B)** Cerebellar Purkinje cell (NMO_10073). **(C)** Striatal SPN (NMO_33253). Normalized difference (ratio) was calculated for both time constants (τ_1_, τ_2_) and steady state (ΔV) to compare modified morphology response to original response. Simulation parameters provided in Methods. Simulation of predictions were quite similar to original morphology for Hippocampal CA1 pyramidal and Cerebellar Purkinje cells, and were not improved by using original initial diameters. Simulation of predictions with original initial diameters improved passive response for the striatal SPN.

**FIGURE 10:**
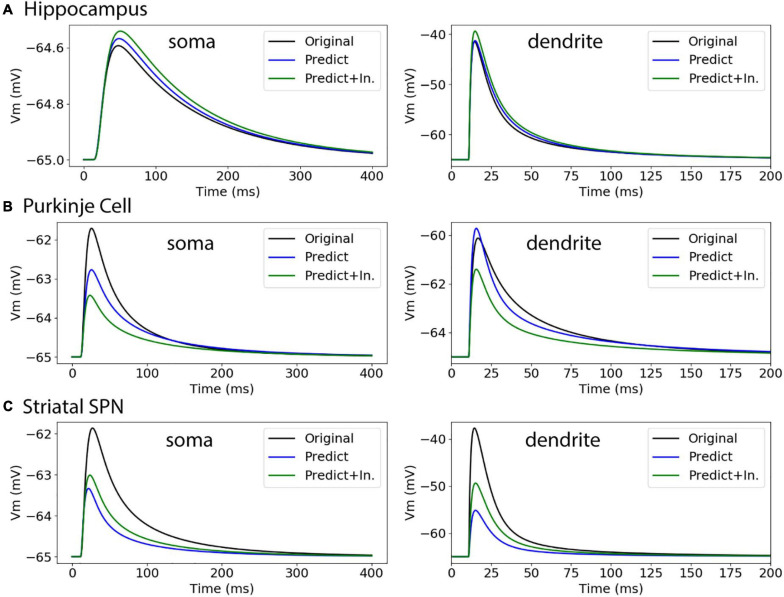
Passive response to synaptic input. **(A)** Hippocampal CA1 pyramidal cell (NMO_35137), with apical and basal dendrites, compartment 32_3. ΔV ratio = 0.009 for dendritic response. **(B)** Cerebellar Purkinje cell (NMO_10073), compartment 12_3. ΔV ratio = 0.082 for dendritic response **(C)** Striatal SPN (NMO_33253), compartment 19_3. ΔV ratio = 0.639 for dendritic response, and improved to 0.427 using original initial diameters.

To further evaluate our predictive diameter equations, we simulated neuron morphologies with predicted and original diameters from the testing set (Table 5 and Figure 9). Simulation of the hippocampal CA1 pyramidal cell (NMO_35137) and the cerebellar Purkinje neuron demonstrates that the passive response of predicted diameters has similar time constants, τ, and steady state, ΔV, to the original morphology. Predictions using the original initial diameters did not improve τ and ΔV for these neurons (Figure 9 and Table 5). The striatal SPN (NMO_33253) simulation had similar τ, but quite different ΔV, and the predictions using original initial diameters improved passive response ΔV (Figure 9). We also simulated the response to synaptic input, measuring both the dendritic response and the somatic response. Figure 10 shows that the synaptic response of predicted diameters was similar to that of original diameters for both hippocampal and cerebellar neurons, especially in the dendrite. The synaptic response of the striatal SPN was greatly improved by using the original initial diameter, but the response was still quite different from the original morphology. In summary, simulation of the passive response to current injection and synaptic input shows a good match to the original morphology when the predicted diameters are moderately correlated with the original diameters.

**TABLE 5:** Passive response for select archive morphologies in test set.

	Test set			Independent morphologies	
Passive response	Hippocampal CA1 pyramidal (NMO_35137)	Cerebellar Purkinje (NMO_10073)	Striatal SPN (NMO_33253)	Hippocampal CA1 pyramidal, golding (NMO_00886)	Striatal SPN, Lindroos (NMO_08390)
*Original τ_1_, τ_2_ (ms)*	21.0, 1.57	21.3, 1.99	21.0, 2.13	23.1, 1.76	15.8, 1.50
*Predict τ_1_, τ_2_ (ratio)*	0.042, 0.035	0.127, 0.025	0.061, 0.117	0.092, 0.171	0.333, 0.081
*Predict* + *In. τ_1_, τ_2_ (ratio)*	0.037, 0.006	0.193, 0.173	0.064, 0.018	0.120, 0.013	0.084, 0.093
*2.0 Diameter τ_1_, τ_2_ (ratio)*	–	–	–	0.225, 0.102	0.094, 0.425
*Original*Δ*V (mV)*	1.16	2.21	3.07	2.17	1.03
*Predict*Δ*V (ratio)*	0.020	0.111	0.671	0.626	0.005
*Predict* + *In.*Δ*V (ratio)*	0.189	0.470	0.493	0.243	0.354
*2.0 Diameter*Δ*V (ratio)*	–	–	–	0.519	0.586

**FIGURE 11:**
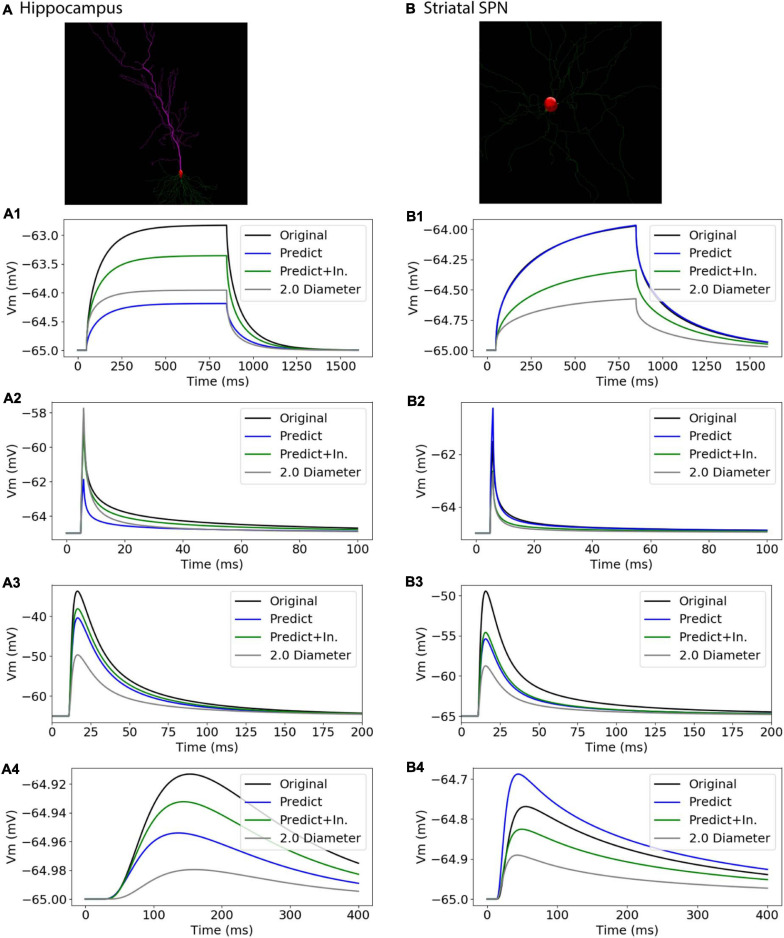
Passive Response with predicted diameters + original initial diameters is similar to that of original diameters for morphologies in validation data. Normalized difference (ratio) was calculated for time constants (τ) and steady state (ΔV) to compare predicted morphology response to original morphology response. **(A)** Hippocampal CA1 pyramidal cell (NMO_00886), with apical and basal dendrites, synaptic input to compartment 31_4. **(B)** Striatal SPN (NMO_08390), synaptic input to compartment 35_3 **(A1, B1)** response to 800 ms somatic current injection. ΔV ratio = 0.24 for hippocampus and 0.35 for striatum for morphologies with original initial diameter **(A2, B2)** response to 1 ms somatic current injection. τ_1_ ratio = 0.12, τ_2_ ratio = 0.01, for hippocampal neuron; τ_1_ ratio = 0.08, τ_2_ ratio 0.09 for striatal SPN for morphologies with original initial diameter. **(A3, B3)** dendritic response to 10 pS. ΔV ratio = 0.14 for hippocampus and 0.33 for striatum for morphologies with original initial diameter. **(A4, B4)** somatic response to 10 pS conductance synaptic input to the dendrite.

*Passive response was calculated for time constant (τ) and steady state (ΔV) for original, predicted, and predictions including original initial diameter (Predict + In.) for selected morphologies (Figure 9). Passive response for morphologies independent of predictive equations (Figure 11) was determined additionally for identical diameter (2.0 Diameter). Original morphology for the Striatal SPN morphology NMO_08390 used predicted diameters from previously reported model equations (Lindroos et al., 2018).*

### Simulation of Independent Morphologies Extend Utility of Predictive Equations

To further assess the utility of predictive equations, we simulated two additional morphologies from distinct NeuroMorpho.org archives not previously used in predictive equations (Table 5 and Figure 11). We also created comparative morphologies with identical dendritic diameter (2 μm) across all nodes, which is the standard corrective procedure if morphologies are submitted to NeuroMorpho.org without explicit diameter. For the hippocampal CA1 pyramidal cell (NMO_00886, Golding et al., 2005) the passive response of the predicted diameter morphology had similar time constant, τ, though different steady state, ΔV. Predictions including original initial diameters did not improve τ, though greatly improved ΔV. The striatal SPN (NMO_08390, Lindroos et al., 2018) was simulated to compare our predictive diameter equations to previously reported predictive diameter equations. Simulation reveals that our predictive equations produce a similar τ and nearly identical ΔV. Predictions using original initial diameters improved τ, but not ΔV. The original striatal SPN (NMO_08390) had diameters of 2.0 μm; thus to provide a measure of how significant these differences are, we simulated the striatal SPN (and the hippocampal CA1 pyramidal cell) using diameters of 2.0 μm. Predicted diameter morphologies better resemble passive response to original morphologies than morphologies with constant dendritic diameter (Table 5 and Figure 11). We also simulated the response to synaptic input, measuring both the dendritic response and the somatic response. Figure 11 shows that the synaptic response using the predicted morphologies is much better than using the 2.0 μm diameters. Using the original initial diameter improved the response for the hippocampal neurons, but not for the striatal neurons. These results suggest that predicted diameters from our model equations are not limited to the select archives used to derive model equations, and may improve utility of available morphologies on NeuroMorpho.org.

## Discussion

We used a combination of morphological features to create predictive diameter equations for multiple neuron cell types: hippocampal pyramidal, cerebellar Purkinje, and striatal SPNs. Separate model equations were created for each of three types of dendritic nodes: initial, branching children, and continuing nodes, to predict diameter from morphological features. Dendritic diameter predictions require PD across cell types, a morphology feature used in a previous predictive diameter model (Lindroos et al., 2018). Additional features, which varied between different cell types, were used to predict diameter for initial nodes and branching children. Predicted diameters of hippocampal pyramidal cells and cerebellar Purkinje cells correlate with original diameters, and simulations reveal similar passive response in these cell types, with improved predictions by including original initial node diameters for striatal SPNs. Simulations of additional morphologies that were independent of the training and testing sets suggest the predictive equations can extend utility to other NeuroMorpho.org morphologies, supplement morphologies without dendritic diameter, and improve model simulations with realistic dendritic diameter.

Further simulation is required to completely assess the response of neurons with predicted diameters. Membrane ion channels modify the response of neurons to synaptic inputs and current injection (Golding et al., 2005; Shah et al., 2010; Debanne and Russier, 2019). Simulations show that variations in channel conductance (Alonso and Marder, 2019) and neuromodulation (Marder, 2012; Marder et al., 2014) can produce drastic differences in neuron activity. This suggests that channel conductance can at least partially compensate for diameter inaccuracies in physiological simulations. We did not present simulations of neurons with active channels to avoid obscuring the role of diameter in controlling passive responses.

Our research continues a long line of studies, beginning with Rall (1962) trying to understand how neuron shape controls activity, as well as investigating what controls neuron shape. Previous studies used morphological features to predict neural branching in growth models (Brown et al., 2008; Donohue and Ascoli, 2008). Cellular processes that control branching may similarly control dendritic diameter. During neurite growth, high tubulin concentration at the soma and high levels of active transport maintain the structural integrity of the growth cone (Hjorth et al., 2014; Mironov et al., 2016; Lanoue and Cooper, 2019). Also, actin is essential to establish, extend, and direct the growth cone toward pre-determined targets (Lanoue and Cooper, 2019). As many neurites grow concurrently, local competition of tubulin and other cytoskeletal components at the soma can selectively increase or decrease dendritic diameter and path length of select branches (Hjorth et al., 2014). The correlation between diameter and path length or branch order may stem from diameter limiting the transport rate of actin and other structural proteins. Additionally, tapering of distal dendrites decreases cellular energy requirements as well as optimizes current transfer along the entire dendritic path (Cuntz et al., 2007; Bird and Cuntz, 2016). Similarly, the extent of dendritic branching can also decrease energy requirements by lowering path length to all terminal ends (Cuntz et al., 2010). Capturing these energy requirements or the addition of microtubules and actin to NeuroMorpho.org (Nanda et al., 2018, 2020) may improve predictions of dendritic diameter.

A possible limitation in the predictive equations is the use of linear regression; however, several analyses suggest that this was not a limiting factor. We used three-dimensional plots to graphically analyze relationship of diameter to morphological features. We assessed whether using the 3/2 power rule at branch children (Rall, 1962) or whether other non-linear feature transformations (e.g., logarithm, power law) would improve predictions of diameter. These extended feature analyses did not improve correlation of features to dendritic diameter for either initial, branching children, or continuing nodes. In summary, multiple linear regression using a combination of features was found to better predict diameter than the non-linear or transformed features. Nonetheless, other non-linear transformations could improve diameter predictions; however, there currently lacks a systematic method to test all possible non-linear relationships or data transformations without considerable involvement. Methods exist to automate non-linear data transformations, such as Artificial or Deep Neural Networks; however, a difficulty remains with interpretation of the mechanisms behind these relationships (Tavallali et al., 2017). Machine learning to automate feature creation (feature engineering) has provided novel insight to proteomics (Ofer and Linial, 2015; Sumonja et al., 2019) and brain connectomics (Pu et al., 2015), suggesting that these approaches may help with predicting dendritic diameter.

The process we utilized to derive predictive equations is not influenced by the accuracy of the dendritic diameters; however, the parameters of the predictive equations are controlled by the diameter values, which may be biased by several factors. One factor is using light microscopy for reconstructions. Light microscopy is limited by light diffraction to a resolution of 0.2 μm (Lu, 2011); thus, dendritic diameter, especially for thin processes, may be over-estimated due to the resolution limit of light microscopy. For example, comparison of the CA1 neuron diameters with electron microscopy diameters (Megias et al., 2001) suggests that the diameter of the thin processes in distal radiatum and lacunosum-moleculare are a bit too large. In addition, the resolution limit of light microscopy may account for the discretized node diameter and the observation that many nodes have diameter equal to PD across our archive morphologies (as shown in insets of Figure 2). A second factor is shrinkage caused by tissue fixation, especially in the z direction. Other considerations with light microscopy such as microscope optics and cell mounting, including magnification and tissue depth, can influence perception of dendritic diameter and diameter-dependent features when tracing neurons (Scorcioni et al., 2004). Thus, alternative imaging techniques are used to overcome current limitations in light microscopy. Electron microscopy can reveal cell ultrastructure to a resolution of 2–4 nm (Lu, 2011) for various brain regions (FitzGibbon and Nestorovski, 2013; Firmin et al., 2014; Liewald et al., 2014), though its utility for large scale neuron reconstructions is limited by acquisition and processing speed of imaging data (Lu, 2011; Silvestri et al., 2013). Newer methods, such as super-resolution imaging (e.g., Stimulated Emission Depletion), can reveal small changes in neural diameter (Chéreau et al., 2017) to a theoretical resolution of 10 nm (Huszka and Gijs, 2019). Another possibility is correlative light electron microscopy, which may be able to provide high resolution diameter estimates of the same neurons being reconstructed (Begemann and Galic, 2016). In the future, applying our method to complete morphologies reconstructed with these methods may improve the predictive equations.

Future extensions to our method could involve integrating diameters measured with newer microscopy methods as well as deriving additional features, e.g., from imaging of cytoskeletal components. Another possibility is to further subdivide dendrites by type of node, e.g., including terminal nodes or branch parents, or according to their location, e.g., within layers in the cortex or hippocampus, because different rules may govern the growth of dendrites in different locations. This may require changes to the reconstruction software, which currently classifies processes into only four classes: soma, axon, apical dendrite, and basal dendrite. A simpler solution, with low processing requirements and using current imaging techniques, would be for reconstructions to include initial node diameters, and then utilize predictive equations to estimate remaining branching children and continuing nodes in the morphology. Providing initial node diameter is practical with standard imaging techniques as initial nodes are physically larger and proximal to the soma in contrast to thin, tapering, and distant dendritic processes. Though manual processing and reconstruction of neuronal morphology remains necessary, predictive equations can provide an alternative method to supplement realistic diameter values and shorten image processing needs using available morphology data. Another approach could be to use electron microscopy or super-resolution imaging of “representative” dendrites from different neuron classes, to derive general equations describing dendritic tapering of continuing nodes which then could be applied to NeuroMorpho reconstructions. These predictive equations would help supplement dendritic diameters that are difficult to capture due to small size, and extend the utility of neuron reconstructions for use in physiology simulations. In theory, our approach is applicable to axons. The predictions of diameter for continuing nodes likely would be different, and possibly better, as axons are not known to taper. On the other hand, the diameters of axons tend to be small, making it difficult to find accurate diameter estimations from light microscopy reconstructions. Ideally, predictive equations could utilize spatial aspects captured by features of original morphology and supplement dendritic diameters across archives within neuron cell types, improving simulations with realistic dendritic diameter for many cell types.

## Data Availability Statement

The datasets analyzed for this study can be found in http://neuromorpho.org/. The neurons can be downloaded using the archive name in Table 1 entered into metadata search by archive. The list of swc files used is provided at http://github.com/neurord/ShapeShifter.

## Author Contributions

JR: morphology selection and analysis, modeling software development and analysis, model simulation and analysis, and manuscript preparation. KB: modeling software development and analysis, model simulation, and manuscript preparation. Both authors contributed to the article and approved the submitted version.

## Conflict of Interest

The authors declare that the research was conducted in the absence of any commercial or financial relationships that could be construed as a potential conflict of interest.

## Footnotes

1 https://moose.ncbs.res.in

2 https://github.com/neurord/ShapeShifter

3 https://bitbucket.org/btorb/btmorph.git

4 https://statsmodels.org
