# Supplementary material for: Prediction of Neural Diameter From Morphology to Enable Accurate Simulation
Source: Front Neuroinform. 2021 Jun 3;15:666695. doi: 10.3389/fninf.2021.666695 (PMC8209307; doi:10.3389/fninf.2021.666695)
Supplement: Supplementary file 1 [file Data_Sheet_1.docx]

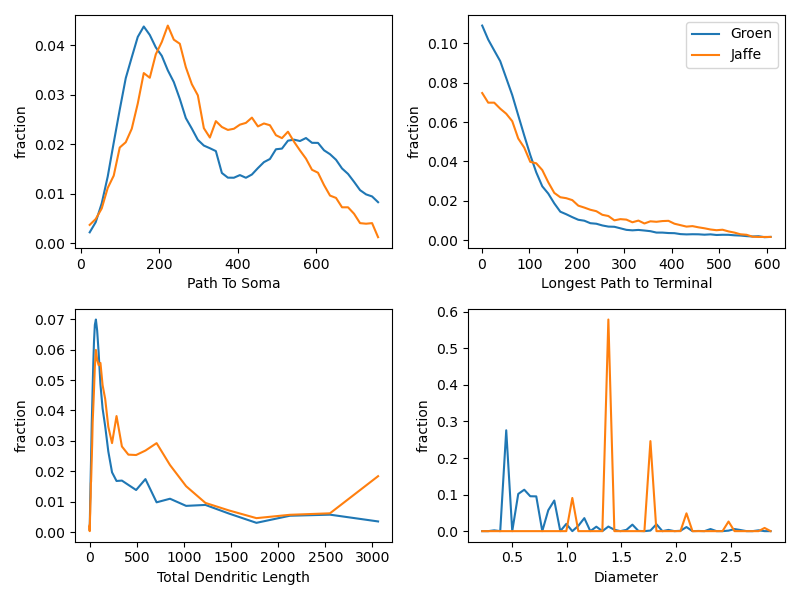
Supplemental Figure 1. Histograms of feature values for Hippocampal Apical dendrites.


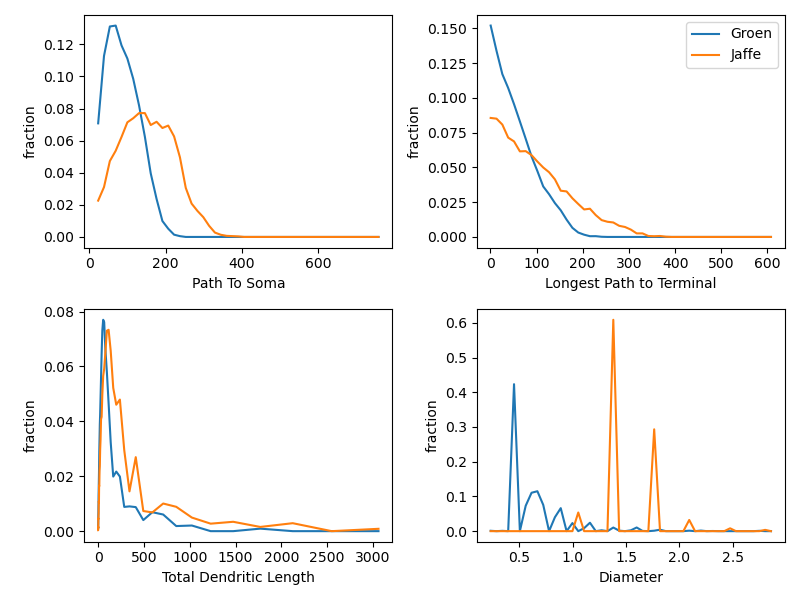
Supplemental Figure 2. Histograms of feature values for Hippocampal Basal dendrites.


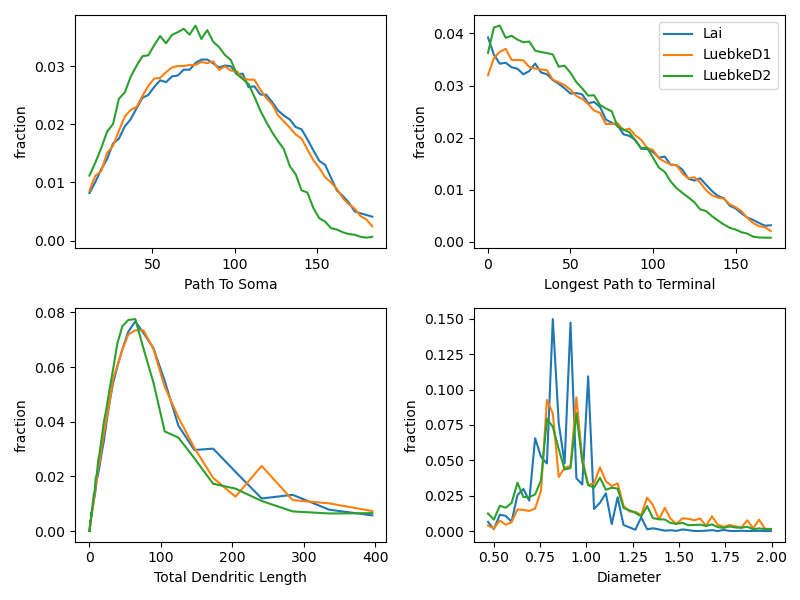
Supplemental Figure 3. Histograms of feature values for Striatal SPN dendrites.


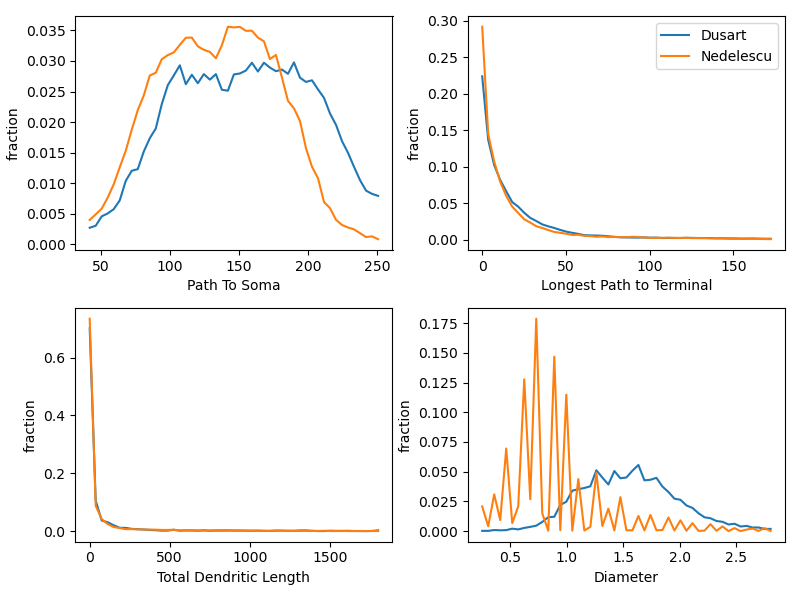
Supplemental Figure 4. Histograms of feature values for Cerebellar dendrites.
